# Screen Time at 6 Years Old and Visual Function in Early Adolescence

**DOI:** 10.3390/vision7040063

**Published:** 2023-09-23

**Authors:** Mathilde Champagne-Hamel, Christine Monfort, Cécile Chevrier, Dave Saint-Amour

**Affiliations:** 1 Department of Psychology, Université du Québec à Montréal, Montreal, QC H2V 2S9, Canada; champagne-hamel.mathilde@courrier.uqam.ca; 2 Inserm, EHESP, Irset—UMR_S 1085, Université Rennes, F-35000 Rennes, France; christine.monfort@univ-rennes.fr (C.M.); cecile.chevrier@univ-rennes.fr (C.C.); 3 Research Center, Centre Hospitalier Universitaire Sainte-Justine, Université de Montréal, Montreal, QC H3C 3J7, Canada; 4 Department of Ophthalmology, Université de Montréal, Montreal, QC H3C 3J7, Canada

**Keywords:** screen time, contrast sensitivity, color vision, acuity, development, children

## Abstract

Excessive screen time has been linked to adverse health outcomes in children, including vision-related problems such as myopia. However, very few studies have evaluated the effect of moderate screen exposure on the development of visual functions. This study aimed to examine the association between screen time during middle childhood and color discrimination, contrast sensitivity, and short-range visual acuity in 12-year-old children (*n* = 305) from the mother–child PELAGIE cohort (France) for the whole sample and for boys and girls separately. Visual functions were assessed using the Freiburg Acuity and Contrast Test and an adapted version of the Cambridge Color Test. Screen exposure was documented using a parent self-report questionnaire. Regression models showed that screen exposure at 6 years of age was significantly associated with higher contrast sensitivity across the entire sample at 12 years of age. However, when controlling for covariates, this association remained statistically significant in girls only. Sex-stratified analyses also showed that moderate screen exposure was linked to improved tritan-axis color vision in boys only. These findings suggest that moderate screen exposure in middle childhood is not harmful to visual function development and as such, provide new insights into the impact of digital technology on children’s visual health and development.

## 1. Introduction

The rise of digital technology has had a significant impact on our daily lives, with children being no exception. With easy access to screens, children are spending more time on digital devices than ever before. Several studies have documented a significant increase in screen time among children and teens, raising concerns regarding the potential adverse effects on their health and development [1,2,3]. As a public health response, several pediatric societies worldwide recommend that children limit their recreational screen time to less than 2 h per day. Additionally, they advise complete avoidance of screen time for children under the age of 2 years, as a critical window of vulnerability has been identified during this period [4,5]. However, children may remain vulnerable to the effects of screen exposure throughout their development. Despite these guidelines, studies conducted prior to the COVID-19 pandemic revealed that just over half of school-aged children adhered to them [5,6]. In France, a comprehensive longitudinal study [7] revealed that children at the age of 5.5 spend an average of 1 h and 34 min in front of screens, with more than three-quarters of children at this age following the public health guidelines [7].

Research suggests that excessive screen time can have detrimental effects on the health of children and adolescents [1,8]. Prolonged screen exposure can reduce sleep quality and a variety of skills (e.g., cognitive, social, and linguistic), as well as contribute to obesity [8,9,10]. Furthermore, screen time can lead to vision impairments, such as eye strain, dry eyes, eye fatigue, and blurred vision. The latter are collectively known as computer vision syndrome [11,12]. Many studies have also associated indoor screen time, close screen viewing, or lack of outdoor time with the prevalence of childhood myopia or nearsightedness [13,14,15,16,17]. Children who spend more time indoors (notably when associated with time spent in front of screens) are more likely to develop myopia than those who spend more time outdoors [18,19].

Various mechanisms have been proposed to explain the potential negative effects of screen exposure on visual health. Exposure to blue light emitted by screens may cause retinal damage, oxidative stress, and inflammation and in turn, impair visual function [20,21,22]. Additionally, vision may be affected given that prolonged screen time may lead to changes in the shape of the lens in the eye, its ability to focus, and changes in the neural processing of visual signals [23,24,25]. Some studies have also suggested that increased time spent indoors and on screens may contribute to a lack of exposure to natural light, which has been shown to have beneficial effects on the eyes. This hypothesis suggests that a lack of natural light exposure may impair the development of the visual system, leading to an increased risk of myopia and other vision problems [26,27,28].

Despite the extensive literature documenting the negative effects of excessive screen time on children’s health and development, very few studies have evaluated whether moderate screen exposure impacts visual functions in children. This research gap is particularly significant considering that moderate screen exposure represents the reality for the vast majority of young individuals. Therefore, our objective in the present epidemiological observational study was to bridge this gap in the literature by investigating the associations between screen time at 6 years of age and general visual functions, namely short-range visual acuity, contrast sensitivity, and color vision, in 12-year-old children from the mother–child PELAGIE cohort in France.

## 2. Materials and Methods

### 2.1. Participants

The PELAGIE (Perturbateurs endocriniens: Étude Longitudinale sur les Anomalies de la Grossesse, l’Infertilité, et l’Enfance) cohort is a longitudinal mother–child cohort that included 3421 pregnant women between May 2002 and February 2006 in Brittany (France). Gynecologists and obstetricians enrolled the women during a consultation in the early stages of pregnancy (before 19 weeks of gestation). The women’s socioeconomic, occupational, and medical characteristics were documented upon inclusion in the study. Information about the pregnancy and the newborn’s health (e.g., birth weight, length, and head circumference) was collected at birth by midwives and pediatricians. Questionnaires were administered when the children were 6 and 12 years old to obtain further information about the child’s development and health, the child’s screen habits, and lifestyle (e.g., participation in sports and nutritional habits).

**Figure 1:**
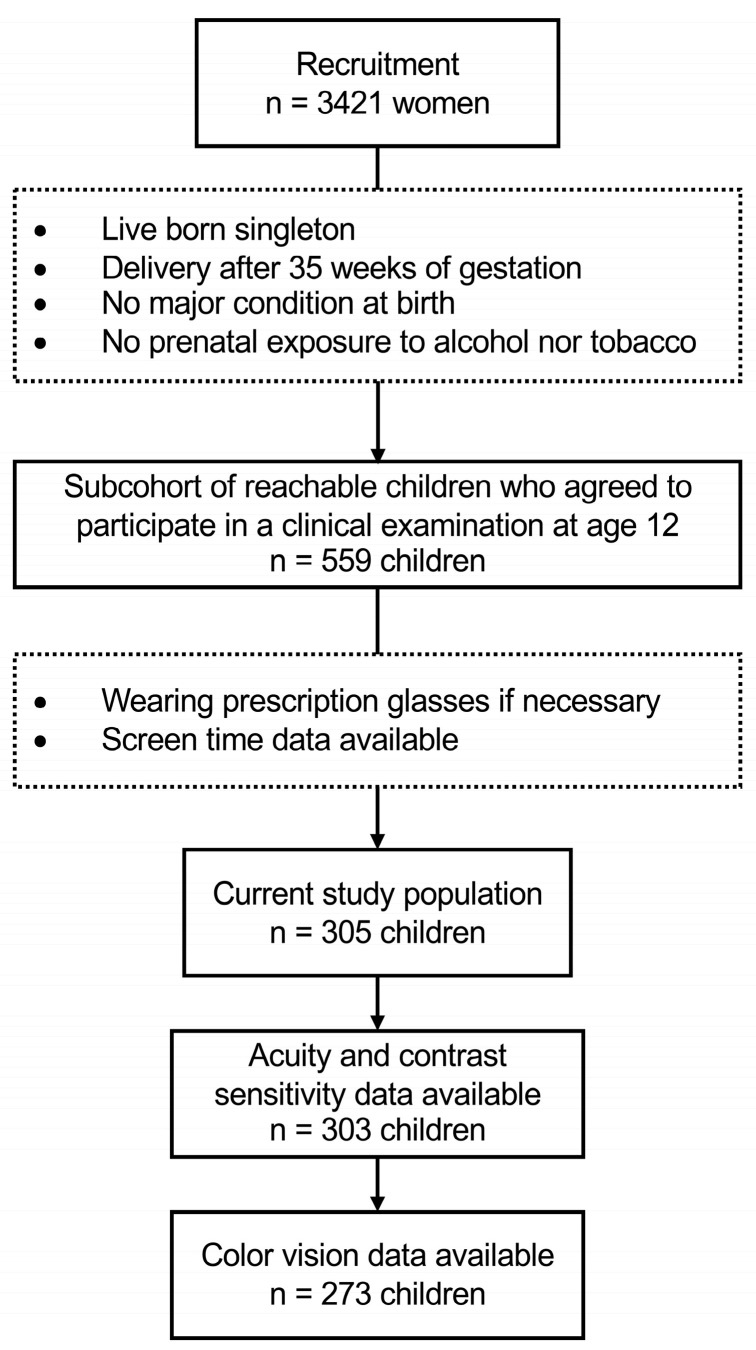
Flow chart for recruitment and inclusion.

By the time the children reached the age of 12, approximately 42% of the families had become untraceable. Thus, for this age point, a total of 1191 families were eligible for the scheduled clinical examination. Of these families, 933 (78.4%) were successfully contacted by phone and among them, 559 (59.9%) children agreed to participate in a clinical examination held at Rennes University Hospital (CHU Rennes). This examination involved various visual tests to assess their vision health. Measures of screen time collected at 6 years of age were available for 319 of the 559 children participating in the clinical 12-year-old follow-up study. Among them, 14 children did not wear their prescription glasses during visual testing and were thus excluded. A comprehensive flow chart for recruitment and inclusions is shown in Figure 1.

The present study was approved by the French Consulting Committee for the Treatment of Information in Medical Research, the French National Commission for the Confidentiality of Computerized Data, and the Université du Québec à Montréal Ethics Committee. Informed consent was provided by the biological mother at recruitment and the primary caregiver at follow-ups with the child. The children provided verbal and witnessed assent.

### 2.2. Measure of Screen Time

In the 6-year and 12-year follow-up studies, participating parents and children completed a questionnaire documenting the approximative amount of time children spend playing video games and watching television per week. Of note, Wednesday was distinguished from the other weekdays (as children in France do not have school on Wednesday afternoon) and the weekend (Saturday and Sunday). Typical weekly screen time was estimated using the following two items: “In a typical week, how much time did your child usually spend watching television or videos?” and “In a typical week, how much time did your child usually spend playing video games (e.g., Computer games, PlayStation, Wii, XBOX)?”. Screen time was quantified by summing the scores for each of these items. Given that screen time is more likely to be harmful to the developing visual system of early-school-aged children, we primarily used screen time measured at 6 years of age for the purpose of the study. That said, the potential influence of screen time exposure measured at 12 years of age was verified in a sensitivity analysis.

### 2.3. Measure of Visual Functions at 12 Years of Age

#### 2.3.1. Visual Acuity and Contrast Sensitivity Threshold

Visual acuity and contrast sensitivity were measured using a modified simulation of the Freiburg Acuity and Contrast Test (FrACT). In this system, Landolt C-like targets are presented as a black or grey symbol on a lighter background in one of four orientations. The participant used the arrows on a keyboard to indicate the target’s orientation as seen on the computer monitor. This assessment included two experiments involving the acuity and contrast components of the test. In both experiments, the progression of optotypes (i.e., the difficulty of the task) was determined using the Best Parameter Estimation by Sequential Testing (PEST) procedure depending on the participant’s answer (correct/incorrect). The contrast test estimated the contrast threshold by modulating the Landolt C-like target luminance level across trials. If the participant responded correctly, the contrast was reduced in the subsequent trial by increasing the optotype luminance. In the visual acuity component, the target size changed with every trial. Indeed, the target shrunk across trials when the subject responded correctly. At the end of each 30-trial test run, the participant’s visual acuity and contrast sensitivity thresholds were recorded for a data analysis as LogMAR and LogCS units. To standardize the testing conditions, the assessments were conducted in similar examination rooms. The subject viewed the stimuli binocularly on a monitor driven by an Apple MacBook Pro (13-inch, Mid 2009). The monitor was placed at a distance of 1.75 m from the participant.

#### 2.3.2. Color Discrimination Threshold

The Ishihara test was first used to screen participants with congenital color deficiencies. The test consists of 17 plates, each illustrating a different color-defined number embedded within other colored dots. The numbers are correctly recognized by people with normal color vision but are not discernible to color-deficient observers. The Ishihara plates were viewed at a distance of approximately 70 cm. The remaining participants were tested using an adapted version of the Cambridge Color Test (CCT; Cambridge Research Systems Ltd., Rochester, UK), which was designed using Psykinematix software version 1.5 (KyberVision Japan LLC, Sendai, Japan). The stimuli were presented using an Apple MacBook Pro (13-inch). Gamma correction was performed using a Spyder5-Pro display calibrator (Datacolor, Trenton, NJ, USA). Chromatic discrimination along dichromatic confusion lines (namely the protan, deutan, and tritan axes) was measured. The assessment involved the presentation of a Landolt C-like target in four orientations against a background that differed in chromaticity. Both the background and target consisted of several discs, each with its own luminance. The observer was instructed to use the arrows on a keyboard to report the orientation of the presented C-like ring. With each correct response, the saturated chromaticity of the target transitioned towards a chromaticity closer to that of the background. The participant had 6 s to respond in each trial. The test used a dynamic staircase method to measure threshold discrimination. The protan, deutan, and tritan staircases were alternated randomly. After 11 reversals for each staircase, color discrimination thresholds were computed as the average vector length and expressed in CIE *u’*, *v’* coordinates. In this task, a lower score (threshold) means a higher discrimination performance. The children viewed the stimuli binocularly from a distance of 1.75 m in a dimly lit room (meso-photopic condition) to ensure that no other light source affected the contrast of the visual simulation on the computer screen.

### 2.4. Potential Covariates

Several potential covariates were documented at the moment of inclusion in the study and at birth: mother’s age, education level (primary/secondary school, undergraduate, or graduate), relationship status, tobacco and alcohol use during pregnancy (yes/no), gestational age, as well as sex and head circumference of the child at birth and the number of children the mother had given birth to before (0 or ≥1). The 6-year and 12-year follow-up questionnaires measured additional variables: breastfeeding (yes/no), sleep duration, weekly physical activity time, wearing of vision glasses, dietary habits, and body mass index (BMI). All variables that were correlated with screen time at 6 years old and with scores for at least one of the visual functions (with a significance level of *p* < 0.2) were included as covariates in the statistical analyses. As a result, sex, head circumference at birth, and BMI measured at the time of testing were included as covariates. Weekly physical activity time was also found to be correlated with both screen time at the age of 6 and at least one of the outcomes. However, as the data for weekly physical activity were limited (available for only 261 participants), we conducted a sensitivity analysis to assess the potential influence of this variable on the results instead of directly incorporating it into the regression models. The same set of covariates was used in all statistical models.

### 2.5. Statistical Analyses

Two-step hierarchical linear regression models (utilizing standardized beta coefficients) were employed to assess the relationship between screen time and children’s FrACT and color discrimination scores. The protan, deutan, and tritan color vision axes, visual acuity scores, and contrast sensitivity scores were considered in separate regression models. Screen time at the 6-year follow up was first entered into the model (unadjusted model). Given that they were found to be potential covariates, sex, head circumference, and BMI were entered into the second block (adjusted model). All analyses were conducted using IBM SPSS software, version 27.0.1.0. (IBM, Armonk, NY, USA). Visual inspection of Q-Q plots of the residuals confirmed that the assumptions of linearity and normality of the residuals were respected. The standardized residuals from each model were examined using scatterplots. No multivariate outliers (defined as an absolute value of standardized residuals >3) were identified. A log2-transformation was used for variables that were not normally distributed (notably for those with positive skewness). Results were considered statistically significant if *p* < 0.05.

## 3. Results

The study sample included 305 participants (129 females) who were 12 years of age (M = 12.81, SD = 0.14). The descriptive characteristics of the participants are reported in Table 1. The age of the mothers at the beginning of their pregnancies ranged from 20 to 43 years old and most were highly educated (completed post-secondary education: 75.7%). The woman gave birth mainly to boys (57.7%), with an average head circumference of 34.7 cm. The average gestational age was 39.6 weeks (ranging from 35 to 42 weeks) and most children were breastfed for at least 2 months. At 12 years old, the children had a mean BMI of 18.1 and engaged in physical activity an average of 3.5 h per week.

Measures of screen time (television, video, and computer) are presented in Table 2 for all children and for girls and boys separately. A *t*-test revealed that screen time did not differ significantly between sexes (*p*s > 0.2). Mean weekly screen time was 8.3 h for all children at the 6-year follow up and 15.9 h at the 12-year follow up. The Pearson correlation between both ages was 0.465 (*p* < 0.001).

Some participants were excluded from the regression analyses for several reasons. Firstly, the FrACT scores of 2 participants and the color discrimination scores of 14 participants were either incorrectly recorded or invalid. Second, 18 additional participants (including 16 boys) were excluded as they made six or more errors on the first 17 plates of the Ishihara test (i.e., indicative of potential congenital color vision deficiencies). It is worth mentioning that the failure rate on the Ishihara test fell within the expected range. This aligns with the understanding that a certain percentage of the population, particularly males, exhibits varying degrees of color vision deficiency.

Descriptive characteristics of FrACT and color discrimination scores for the remaining participants are presented in Table 3. Visual functions did not differ significantly between sexes, apart from color vision in the tritan axis where boys had significantly better scores than girls (0.996 vs. 0.927, *p* = 0.004). The mean color discrimination thresholds of the whole sample for the protan, deutan, and tritan thresholds were 0.499, 0.184, and 0.958, respectively (in *u’v’* units). The mean contrast sensitivity threshold was 1.941 log units, while the mean visual acuity threshold was −0.211 logMAR (Snellen equivalent of 1.5).

The results of the regression analyses examining the relationship between screen exposure assessed at 6 years old and the FrACT and color discrimination scores assessed at 12 years old are shown in Table 4, Table 5 and Table 6 for the whole sample, as well as for boys and girls separately. When considering the whole sample, a significant association was observed between the screen time and contrast sensitivity threshold (β = −0.126, *p* = 0.029) (unadjusted model). After adjusting for covariates, this association became non-significant (β = −0.110, *p* = 0.060). No other statistically significant associations were found between the screen time and color vision threshold or visual acuity. Among boys, no significant association was observed between screen exposure and visual acuity, achromatic contrast sensitivity, or color discrimination in the protan and deutan axis. However, a significant association was found between the screen time and color vision threshold in the tritan axis (β = −0.171, *p* = 0.042) after controlling for potential covariates (adjusted model). Among girls, higher screen exposure was significantly associated with a lower achromatic contrast sensitivity threshold (β = −0.282, *p* = 0.001). This association remained statistically significant in the adjusted model (β = −0.254, *p* = 0.004).

A first sensitivity analysis was conducted by re-running the regression models without participants wearing glasses at the time of the visual testing (*n* = 83), i.e., without the participants with correction for refraction error (44 of the 83 participants were myopic according to parent report). Results revealed no substantial change (<10% in the β coefficients) in the associations reported above. Furthermore, screen time exposure in participants wearing glasses was not statistically different from other participants (*p*s > 0.1) A second sensitivity analysis was performed by adding weekly physical activity time to the regression models; no substantial change (<10% in the β coefficients) in the results was observed. Finally, a third sensitivity analysis was conducted by adjusting the models for screen time exposure at age 12; results showed no substantial change (<10% in the β coefficients) in the associations.

## 4. Discussion

In this study, we investigated the relationship between screen exposure at an early school-age period and later visual functions in 12-year-old children. We utilized the Freiburg Vision Test to assess contrast sensitivity and short-range visual acuity and an adapted version of the Cambridge Color Test to assess color discrimination. Despite the absence of established clinical norms for these tests, the mean scores recorded for our sample (individuals with normal or corrected-to-normal vision) fell within the expected range for this age group [29,30].

Regression analyses conducted on the whole sample revealed that screen exposure at 6 years of age was associated with higher sensitivity to achromatic contrast. However, this association became non-significant when controlling for covariates. When the sample was stratified by sex, we observed different result patterns for boys and girls. Specifically, no significant associations were found between screen time and short-range visual acuity, achromatic contrast sensitivity, or color vision in the protan and deutan axes for boys. However, a significant association (in both the unadjusted and adjusted models) was observed between screen time and improved color vision in the tritan axis. Among girls, a strong association was found between screen time and achromatic contrast sensitivity (in both the unadjusted and adjusted models).

Contrary to our initial expectations, our study yielded results that challenge the prevailing belief regarding the detrimental impact of screen exposure on visual function. Indeed, our findings not only suggest an absent association between screen exposure and a decrease in the examined visual outcomes but that improvements can be observed in some sex-specific cases. These results diverge from previous studies that primarily focused on refractive errors (e.g., myopia or visual acuity) as the key indicators of visual health. Of note, our study’s primary objective was to investigate the relationship between screen time at the age of 6 and broader aspects of visual function under normal or corrected-to-normal refraction viewing conditions. Said differently, we intended to extend beyond the traditional outcomes of myopia and acuity impairment that have been previously reported in the literature. Furthermore, it is important to highlight that our study did not find any association between the need to wear glasses and screen exposure.

One of our novel and intriguing findings is the association between screen exposure and increased color discrimination in the tritan axis. Although the underlying mechanisms are unclear, exposure to blue light emitted by screens may stimulate the retinal cells responsible for color perception and ultimately enhance their sensitivity [31,32,33,34]. Thus, moderate exposure to blue light from digital screens could have a greater impact on the tritan axis (compared to the other axes) due to the higher sensitivity of the corresponding blue-light photoreceptor cells to short-wavelength light (i.e., blue light). However, this association was observed exclusively among boys. One hypothesis for this gender-specific effect is related to potential differences in visual processing and sensitivity to blue light between the sexes. It is known that there are inherent variations in retinal physiology and visual system development between males and females [35,36]. These differences may influence how the retinal cells respond to blue light exposure and subsequently impact color discrimination in the tritan axis. Further research is needed to explore and confirm this sex-specific effect and uncover the underlying contributing factors.

Another intriguing finding of our study is the association between increased screen exposure and enhanced contrast sensitivity. As mentioned earlier, our results indicate that children who reported spending more time looking at screens had better contrast sensitivity than those who reported less screen time. This finding is consistent with previous research that suggests that some visual functions may improve with increased screen time. For example, it has been shown that playing video games may improve spatial and temporal visual processing [37,38,39]. This enhancement of the visual function is thought to rely on the brain’s capacity to adapt and reorganize neural pathways following extensive visual experience. In fact, action video games demand rapid visual processing and heightened attention to detail, fostering neural adaptations in the visual cortex. Through extended gameplay, players appear to develop greater sensitivity to subtle differences in contrast, ultimately improving their ability to discern fine details in complex visual scenes. Moreover, there is evidence to suggest that playing video games can improve attentional control and the ability to selectively attend to relevant information. In turn, the latter could also contribute to enhanced contrast sensitivity [39,40]. Nonetheless, the participants in our study did not report spending a lot of time playing video games at the age of 6. It is therefore challenging to translate the relationship between video game play and contrast sensitivity within the context of our study.

Overall, our findings suggest a moderation effect of sex in the observed association between screen exposure and visual function. To make sense of these findings, some evidence shows sex differences in visual function [35]. While research in this area is still limited and controversial, it has been reported that males tend to have better visual acuity than females, while females tend to have better color discrimination ability [41]. Other studies have found sex differences in visual processing speed, contrast sensitivity, and visual attention, though the results have been mixed and may depend on the specific task and population studied [36,42,43]. Some researchers have proposed that the observed sex differences in visual function may be attributed to biological factors (e.g., sex hormones and brain structure) and sociocultural influences (e.g., gender roles and experiences) [35,36]. Nevertheless, more research is required to fully understand the mechanisms underlying these differences and their implications for visual health and development. Our findings suggest that a more nuanced approach to screen use and visual health may be needed, where future studies should consider individual factors such as age and gender.

The present study had several limitations that should be considered when interpreting the results. One major limitation is that the screen exposure measures for the children in our sample were taken between 2009 and 2012 and therefore may not reflect the screen exposure of children today. With the rapid advances in technology over the past decade, the types of screens, devices, and apps that children use, and the amount of time spent looking at screens, have changed significantly. Therefore, the screen exposure of our participants may not be representative of current screen exposure patterns, which could limit the generalizability of our findings to the broader population of children today. Another limitation of our study is that we relied on parental self-reports to estimate their children’s screen time, which are subject to recall bias and lack of accuracy. However, screen time measures at 6 and 12 years old showed a good correlation (r = 0.47), suggesting some reliability between the two measures. Finally, our study did not investigate the potential negative effects of excessive screen time on children’s visual health, such as refraction error, eye strain, headaches, dry eyes, and other visual discomforts. While our results did not suggest an association between screen exposure and decreased visual function, it is important to consider both the potential benefits and risks of screen use when making recommendations for optimal visual health in children.

Despite these limitations, our findings provide some evidence to suggest that moderate screen exposure in children is not associated with harmful effects on vision. This represents a significant contribution to the literature given that concerns about the negative effects of screen time on children’s health have been widely discussed in recent years. Further research, including studies with an experimental design, is needed to explore the potential long-term effect of screen exposure on visual function and to investigate the mechanisms underlying the associations documented in this study.

## Figures and Tables

**Table 1:** Descriptive characteristics of the participants (*n* = 305).

	*n*	%	Mean (SD)
Characteristics of the mothers			
Mothers’ age at inclusion	302		30.7 (3.8)
Mothers’ educational level			
≤High school	74	24.3	
>High school	231	75.7	
Characteristics of the children			
Sex			
Male	176	57.7	
Female	129	42.3	
Gestational age	303		39.6 (1.2)
Head circumference	304		34.7 (1.2)
Breastfeeding			
Yes	218	71.5	
No	86	28.2	
BMI at 12 years old	305		18.1 (2.6)
Physical activity (hours/week)	261		3.5 (2.0)

**Table 2:** Descriptive characteristics of screen time (in hours) per week.

	All Children (*n* = 305)		Girls (*n* = 129)		Boys (*n* = 176)	
	Mean	SD	Mean	SD	Mean	SD
Total screen time						
6 years	8.3	4.9	7.9	4.8	8.6	4.9
12 years	15.9	8.2	14.9	8.1	16.7	8.2
Television/movie viewing						
6 years	6.9	4.0	6.7	4.1	7.1	4.0
12 years	10.6	5.8	11.3	6.2	10.0	5.4
Video/computer gaming						
6 years	1.4	1.9	1.2	1.7	1.5	2.0
12 years	5.4	5.1	3.6	4.4	6.7	5.2

**Table 3:** Descriptive characteristics of FrACT and color discrimination scores at the 12-year follow up.

	All Children			Girls			Boys		
	*n*	Mean	SD	*n*	Mean	SD	*n*	Mean	SD
FrACT	303			128			175		
Visual Acuity (LogMAR)		−0.211	0.092		−0.203	0.102		−0.217	0.084
Contrast threshold (Log)		1.941	0.208		1.924	0.219		1.954	0.199
Color discrimination (*u’v’)*	273			121			152		
Protan axis		0.499	0.196		0.515	0.199		0.487	0.194
Deutan axis		0.184	0.333		0.216	0.341		0.159	0.326
Tritan axis		0.958	0.196		0.996	0.179		0.927	0.205

**Table 4:** Associations between screen exposure measured at 6 years of age and the FrACT (*n* = 303) and color discrimination (*n* = 273) scores in the entire sample at 12 years of age.

	Unadjusted Model	Adjusted Model
	β (95% CI)	β (95% CI)
FrACT		
Visual acuity	0.045 (−0.001, 0.003)	0.036 (−0.001, 0.002)
Contrast threshold	−0.126 (−0.010, −0.001) *	−0.110 (−0.009, 0.0002) ^†^
Color discrimination		
Protan axis	0.051 (−0.003, 0.007)	0.028 (−0.004, 0.006)
Deutan axis	−0.002 (−0.008, 0.008)	−0.026 (−0.010, 0.006)
Tritan axis	−0.079 (−0.008, 0.002)	−0.080 (−0.008, 0.002)

Variables included in the adjusted regression model were sex, head circumference at birth, and BMI. ^†^ *p* ≤ 0.10, * p ≤ 0.05.

**Table 5:** Associations between screen exposure at 6 years of age and the FrACT (*n* = 175) and color discrimination (*n* = 152) scores in boys at 12 years of age.

	Unadjusted Model	Adjusted Model
	β (95% CI)	β (95% CI)
FrACT		
Visual acuity	−0.0004 (−0.002, 0.002)	−0.021 (−0.003, 0.002)
Contrast threshold	−0.010 (−0.006, 0.005)	0.003 (−0.006, 0.006)
Color discrimination		
Protan axis	0.007 (−0.006, 0.006)	−0.028 (−0.007, 0.005)
Deutan axis	−0.029 (−0.012, 0.009)	−0.050 (−0.014, 0.008)
Tritan axis	−0.155 (−0.013, 0.0002) ^†^	−0.171 (−0.014, −0.0003) *

Variables included in the adjusted regression model were sex, head circumference at birth, and BMI. ^†^ *p* ≤ 0.10, * p ≤ 0.05.

**Table 6:** Associations between screen exposure at 6 years of age and the FrACT (*n* = 128) and color discrimination (*n* = 121) scores in girls at 12 years of age.

	Unadjusted Model	Adjusted Model
	β (95% CI)	β (95% CI)
FrACT		
Visual acuity	0.115 (−0.001, 0.005)	0.096 (−0.001, 0.005)
Contrast threshold	−0.282 (−0.020, −0.005) **	−0.254 (−0.019, −0.004) **
Color discrimination		
Protan axis	0.130 (−0.002, 0.013)	0.093 (−0.004, 0.011)
Deutan axis	0.057 (−0.009, 0.017)	0.014 (−0.012, 0.014)
Tritan axis	0.106 (−0.003, 0.010)	0.080 (−0.004, 0.009)

Variables included in the adjusted regression model were sex, head circumference at birth, and BMI. ** p ≤ 0.01.

## Data Availability

The dataset used in this current study is available from the corresponding author upon reasonable request.
